# A significant difference of synovial mast cells in synovium from rotator cuff arthropathy compared to rotator cuff tears: A histological pilot study

**DOI:** 10.1016/j.ocarto.2024.100503

**Published:** 2024-07-19

**Authors:** Luca Farinelli, Francesco D'Angelo, Carlo Ciccullo, Sandra Manzotti, Antonio Gigante

**Affiliations:** a Clinical Orthopaedics, Department of Clinical and Molecular Sciences, Università Politecnica delle Marche, Ancona, Italy; b IRCCS INRCA, Ancona, Italy

**Keywords:** Glenohumeral osteoarthritis, Rotator cuff arthropathy, Synovial mast cells, Inflammation, OA

## Abstract

**Objective:**

Aim of the present study was to compare the presence of Mast Cells (MCs) in synovial samples from gleno-humeral osteoarthritis (OA) and from control group.

**Methods:**

Synovial tissue samples were obtained during arthroplasty from 23 patients with gleno-humeral OA due to rotator cuff arthropathy (RCA) and from 20 patients without OA, constituting OA group and control group respectively. Before surgery self-reported pain was assessed using VAS score and OSS was used to value functional ability. Shoulder radiograph (Antero-posterior, Y-view and Grashey views) was evaluated by musculoskeletal radiologist and graded according to modified Samilson-Prieto classification.

Synovial tissue, obtained during arthroplasty and arthroscopic procedure, was prepared to immunohistochemical analysis with anti-CD31 and anti-CD117 antibodies, to detect respectively endothelial cells and MCs at 40x magnification. Synovitis scores have been assessed. Under the control of the image processing system the distribution and the total number of vessels and MCs were determined.

**Results:**

The numbers of MCs and the area fraction (20x magnification) occupied by them were significantly higher in OA samples than in control tissue. The synovitis score was higher in OA patients with a positive correlation. Vessels number and area fraction were higher in OA patients than in controls. Analysis of MC number in relation to clinical data indicated positive correlation with the VAS score.

**Conclusions:**

The distribution of MCs on synovium significantly differ between OA and control groups. Despite the design of the study could not conclude the cause-effect relationship, the presence of MCs might have role in OA pathogenesis.

**Level of evidence:**

Histological study.

## 1 Introduction

Osteoarthritis (OA) is a degenerative disease characterized by changes in articular and peri-articular structures. These changes include cartilage damage, subchondral sclerosis, periarticular bone formation, periarticular soft tissues alterations and synovial inflammation [1].

However, OA is not considered an inflammatory arthropathy since the count of leukocytes in synovial fluid is commonly below the threshold for “inflammatory disorder” [2]. Synovitis plays an important role in OA progression presenting clinically as joint swelling, pain and morning stiffness [3,4]. The association between OA and synovial inflammation has been examined and confirmed in recent years [5], demonstrating how cartilage damage and progression of typical symptoms could be related to the degree of synovitis [6].

Although, little is known about the biological processes that explain this association, it has been suggested that synovitis and immune cells could be active players in OA development and progression [7,8]. The most represented inflammatory cells in synovial tissues are Macrophages, T lymphocytes and Mast Cells (MCs) [8].

MCs are sentinels of the innate immune system residing both in normal and pathologic human joints. They can be predominantly localized in the sub-synovial lining layer and at the junction between cartilage and synovium [9].

MCs can be activated by a variety of different stimuli, including allergens, pathogens, and endogenous mediators, acting on their receptors such as Toll Like Receptors, Immunoglobulin G receptors, SCF/c-kit axis and IgE/FceRI/Syk axis. These receptors induce the release of pre-formed mediators leading to synovial inflammation, angiogenesis and bone destruction [8].

MC activation and degranulation via IgE/FceRI/Syk axis has been reported to promote tissue damage and inflammation in a mouse model of OA, whereas inhibition of FceRI signalling reduced cartilage breakdown, osteophytes formation and synovitis [10,11]. Moreover, MCs mediators have been found on synovial fluid in OA suggesting MCs activation and subsequent degranulation [12].

Furthermore, new insights on the effect of anti-IgE therapy in atopic patients with knee OA have shown improvement of clinical symptoms suggesting that IgE-mediated activation of MCs may play a role [13].

MCs are located in the vicinity of nociceptive fibers and may interact with nerves through synapse-like connections [14]. In fact, these are increased in many conditions characterized by pain, including models of post-fractures nociception and postoperative pain [15,16].

Up to date, MCs are not extensively investigated on their possible role in synovial inflammation and OA pathophysiology.

The primary aim of the present study was to compare the presence and distribution of MCs of synovial samples from gleno-humeral OA from rotator cuff arthropathy (RCA) and to compare it with synovium samples from patients with rotator cuff tear (RCT) that underwent to arthroscopic repair. Secondly, we aimed to correlate MC number with preoperative pain, functional score and the radiological grade of OA.

## 2 Materials and methods

### 2.1 OA patients

Synovial tissue samples were obtained during arthroplasty from 23 patients with gleno-humeral OA from RCA treated at Azienda Ospedaliera-Universitaria Ospedali Riuniti of Ancona (Ancona, Italy) from March 2021 to January 2022.

Three synovial samples were taken, in most significant synovitis area, form each subject.

Patients provided their informed consent to participate in the study. Inclusion and exclusion criteria are summarized in Table 1.

**Table 1:** Inclusion and exclusion criteria of patients with Rotator cuff arthropathy (RCA) and Rotator cuff tears.

Inclusion criteria	Exclusion criteria
Age>18 years old	Rheumatologic diseases
History of pain	Atopy
Limitation of daily living	Diabetes and other endocrinological diseases
Poor response to non-operative management	Previous shoulder surgery and/or fractures
Radiographic joint changes (mSP II and III)^a^	Shoulder infiltration (intrarticular and/or periarticular in the last 6 months)

a RCA group.

### 2.2 Control group

Shoulder synovial tissue was obtained, in the same manner described above from 20 patients without OA, whose mean age was 49.2 (range 32–64), who underwent arthroscopic repair due to traumatic RCT, and define the characteristics of tear according to Patte classification: 16 type 2 (80%); 4 type 1 (20%) and Cofield classification: 11 small tear <1 ​cm (55%); 9 medium tear 1–3 ​cm (45%). None of these had chronic pain before trauma or clinical and radiological evidence of shoulder OA.

### 2.3 Clinical and radiological data

Before surgery, self-reported pain was assessed using the visual analog scale (VAS 0–100), and Oxford Shoulder Score was determined. Standard Antero-Posterior view, Y-view and Grashey view were obtained from participants and graded according to Hamada classification [17] by an experienced musculoskeletal radiologist who was blinded to patient characteristics.

### 2.4 Histological and histomorphometric analysis

Synovial tissues obtained from surgery were fixed in 10% buffered formalin, embedded in paraffin and sectioned to a thickness of 3–5 ​μm.

Sections were dewaxed in xylene and rehydrated through a graded series of ethanol (all reagents Bio-Optica SpA, Milano, Italy).

For routine histological examination, sections were stained with Hematoxylin-Eosin. Superfrost Ultra Plus® slides (Menzel-Gläser, Braunschweig, Germany) were used for immunohistochemical analysis. For immunolabelling with CD31 sections were treated with EnVision FLEX Target Retrieval Solution High pH for antigen retrieval. On all sections endogenous peroxidase activity was inhibited with Dual Endogenous Enzyme Block for 10 ​min at Room Temperature.

Subsequently, sections were incubated with polyclonal rabbit anti-human CD117 antibody and with monoclonal mouse anti-human CD31 antibody, diluted 1:50 and 1:40 respectively in EnVision FLEX Antibody Diluent, in a humidified atmosphere for 20 ​min. For the washes between the various steps Tris-HCl buffer pH 7.6 was used.

The antigen-antibody complex was revealed using the Dako EnVision™ ​+ ​Dual Link System, HRP/DAB, according to the manufacturer's protocol (all reagents Dako Agilent Technologies, Carpinteria, CA, USA). A brown precipitate is generated in the presence of the antigen-antibody binding. Sections were finally counterstained with Mayer's hematoxylin (Bio-Optica SpA, Milano, Italy). Negative control was represented by primary antibody untreated sections. Sections were examined with light microscope Leica Leitz DMRBE (Leica Microsystem, Wetzlar, Germany) equipped with Leica Application Suite digital analyzer (Leica Microsystem, Heerbrugg, Switzerland).

Under the control of the image server, the fields were selected and acquired on the screen. The camera image was digitalized by the image-capture board. The total number of MCs and vessels were determined in 10 adjacent high-power (40x magnification) fields (hpf), in the middle of the tissue section, starting on the left side of the section at the lining layer. Moreover, the area fraction (%) occupied by MCs and vessels were evaluated at 20x magnification in 10 field/specimen. Two independent blinded observers counted MCs and vessels, to assess MCs and vessel area fraction has been used software Leica Application Suite V4.9, checked manually. To assess inflammation, samples were scored according to Krenn et al. and were classified on a scale from no synovitis (0–1), low-grade synovitis (2–4) and high-grade synovitis (5–9) according to synovitis score [18].

### 2.5 Statistical analysis

Statistical analysis was performed using the JMP statistical software. Categorical variables were expressed in. numbers and percentages (%). Continuous variables were expressed by average and standard deviation. The normal distribution of variables was verified through Shapiro-Wilk test. Variables were normally distributed, therefore parametric test was used to detect differences between groups. Specifically, Student's t-test was used. Correlation studies was performed by linear regression analysis, using Pearson's correlation coefficient ρ. Significance was set at p ​< ​0.05. Iterobserver variability was determined with Cohen's kappa coefficient.

## 3 Results

**Fig. 1:**
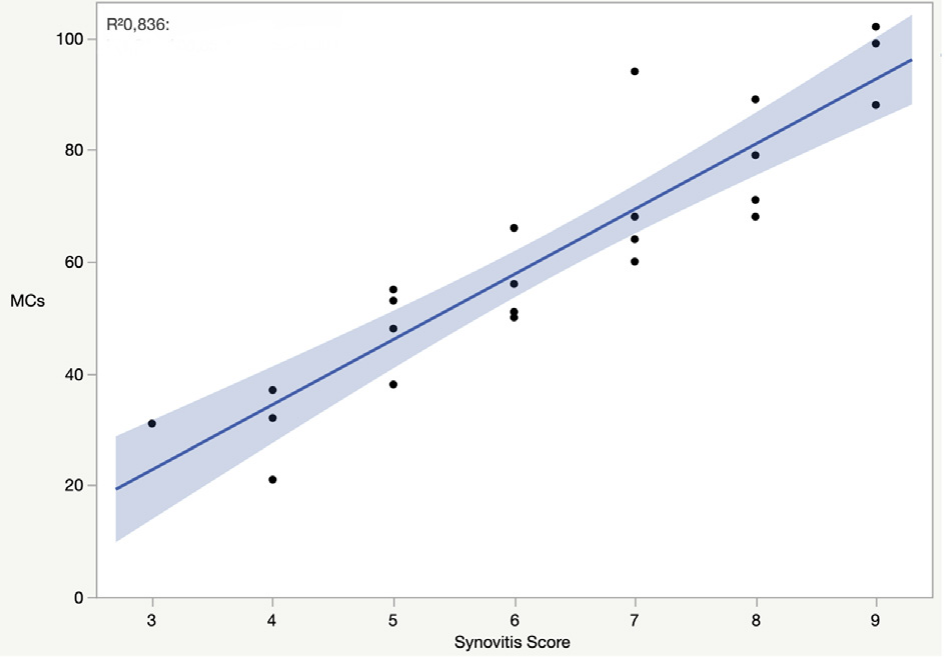
Correlation of synovitis score and Mast Cells (MCs) R^2^= Pearson correlation coefficient.

**Fig. 2:**
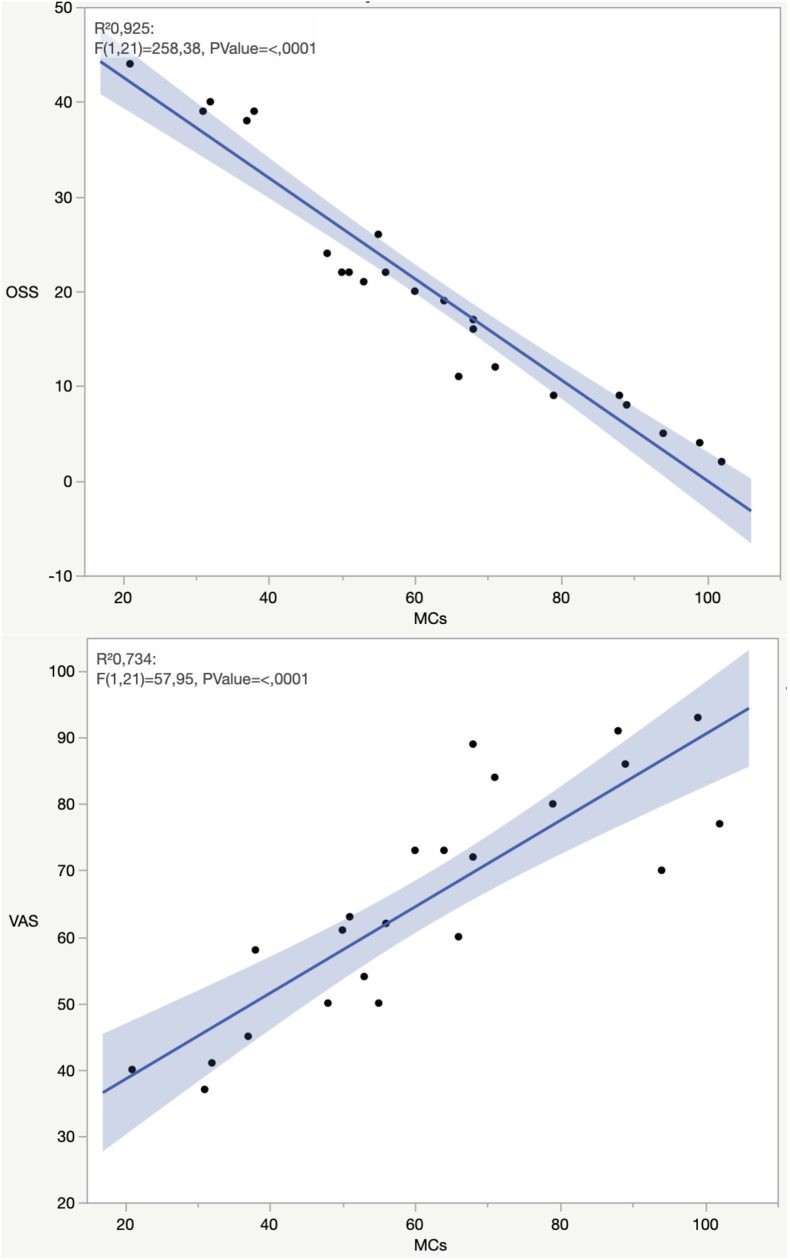
Correlation between MCs and functional scores. R^2^= Pearson correlation coefficient.

The clinical data of all participants are summarized in Table 2. The numbers of MCs and the area fraction occupied by them were significantly higher in shoulder affected by OA than in control subjects (p ​< ​0.05) (k ​= ​0.87) (Table 3). The synovitis score was higher in OA patients. Vessels number and area fraction were higher in OA patients than in controls. Examination of MC number in relation to the degree of synovial inflammation highlighted a positive correlation with the synovitis score (r ​= ​0.914) (p ​< ​0.05) (Fig. 1). Analysis of MC number in relation to clinical data indicated a positive correlation with the VAS score (r ​= ​0.856) (p ​< ​0.05). The examination of MC number in relation with functional shoulder scale (OSS) showed a negative relation between these two parameters (r ​= ​- 0.961) (p ​< ​0.05) (Fig. 2). Lastly, even though the number of MCs was higher in Hamada grade 3/4 than in Hamada grade 1, no significant correlation was found between number of MCs and radiographic stage according to Hamada classification (r ​= ​0.294) (p ​> ​0.05).

**Table 2:** Clinical data of 23 and 20 patients of respectively RCA and RCT group.

	RCA group	RCT group	p-value
Patients	23	20	
Age (years), mean (SD)	71.2 (4.2)	49.2 (6.3)	**0.04**
Female N. (%)	8 (35%)	11 (55%)	n.s.
Hamada classification			NA
grade 2 No. %	7 (30.4%)		
grade 3 No. %	13 (56.5%)		
Grade 4° No. %	3 (13.1%)		
Pain VAS, mean (SD)	65.6 (17.13)	60.3 (8.09)	n.s
OSS, mean (SD)	29.91 (12.12)	40.32 (4.91)	n.s.

RCA: rotator cuff arthropathy; RCT: rotator cuff tear; OSS = Oxford Shoulder Score, SD = standard deviation; n.s.: not significance; NA: not applicable; Bold: statistical significant (p <0.05).

**Table 3:** Mast cells (MCs) infiltrate, synovitis score and vascularity of synovium of RCA and RCT groups.

	RCA group	RCT group	*p*-value
MCs/field (SD)	48,5 (17,21)	18,23 (6,9)	n.s.
Area fraction (SD) (%)∗	0,26 (0,1)	0,11 (0,06)	0.04
Synovitis score (SD)∗	3,34 (1,7)	0,8 (0,27)	0.001
Vessel N. Mean (SD)∗	52 (18,9)	13,7 (6,9)	0.05
Vessel area fraction (SD) (%)∗	2,7 (0,9)	0,61 (0,5)	0.04

SD: standard deviation; ∗ significant differences with *p* ​< ​0.05.

## 4 Discussion

**Fig. 3:**
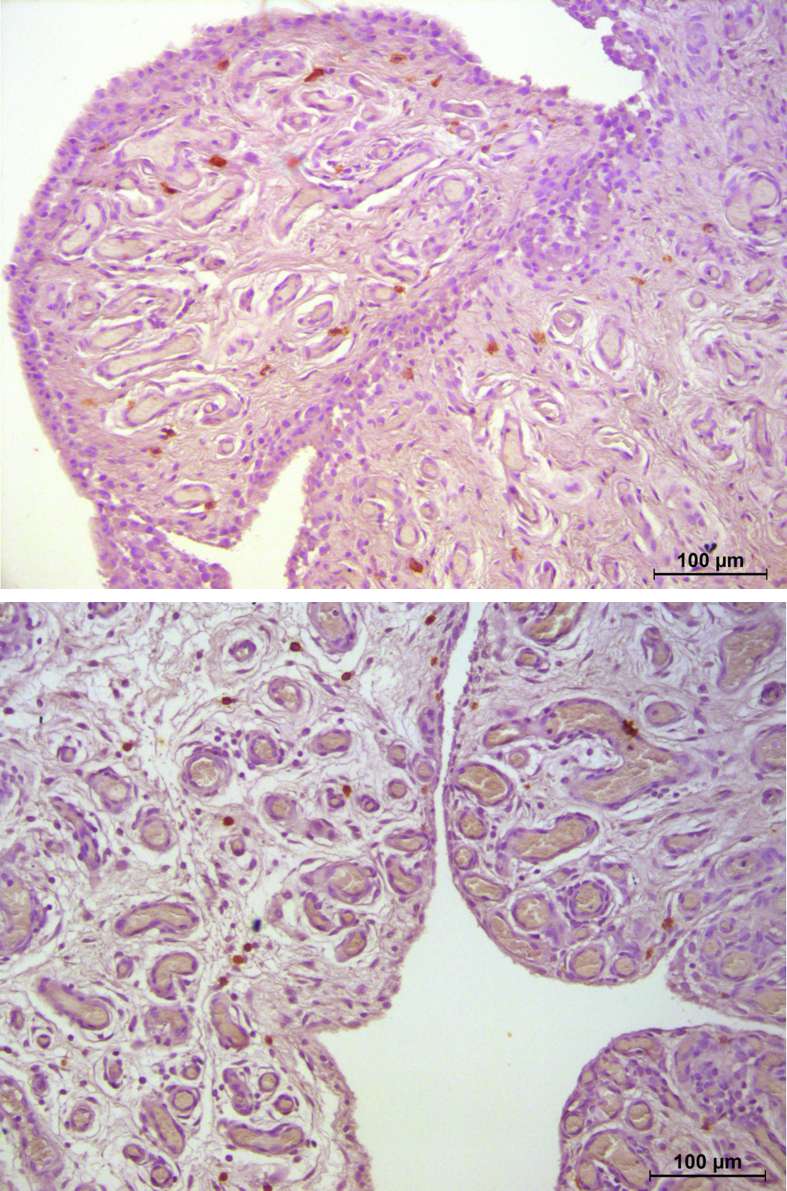
Sample of synovium from Rotator Cuff Arthropathy (RCA) group, paraffine section stained by CD117, (magnitude 20×). Mast cell are identified by brown reaction product.

The main finding of the present study was that the number of MCs of synovium of patients with RCA was significantly higher compared to RCT patients. MCs were predominantly found in the sublining synovial layer in all participants [9] (Fig. 3). MCs and their mediators are known to be found in the synovium and synovial fluid of OA patients [15]. Our findings indicate that a greater number of MCs was positively correlated with higher levels of synovitis and worse preoperative clinical scores. Indeed, previous studies have suggested that stem cell factor secreted by synovial cells could lead to recruitment and hyperplasia of MCs into synovium and this could be associated with histologic inflammation [19]. Recently, in a novel mice model, it has been reported that intraarticular injection of MCs induced cartilage degeneration, increasing pain levels and synovial inflammation [20].

In addition, it has been shown that IgE-FceRI axis is crucial for the development of OA, demonstrating that inhibition of this pathway reduced the onset of histopathological changes related to OA, including osteophytes formation^11^. Sousa Valente et al. demonstrated that MCs tend to cluster in the proximity of nerves fibers in the synovium. Once activated by NGF signaling with a FceRI-mediated pathway, MCs increase prostaglandin D2 release that sensitize nociceptive fibers [21].

Data from our study showed that a higher number of MCs in synovium correlated with increased pain assessed with VAS score. These findings suggest that MCs may play a role in pain perception also in shoulder OA. As we previously reported, similar positive correlation between synovium MCs and clinical and histopathological findings can be demonstrated in hip and knee OA [7], albeit the number of MCs/field and Area fraction tend to be lower in shoulder RCA, probably because it is a non-weightbearing joint or maybe there is a different gene expression, even though controversy still exists on which signaling cascade is to be considered responsible of MCs activation in OA.

**Fig. 4:**
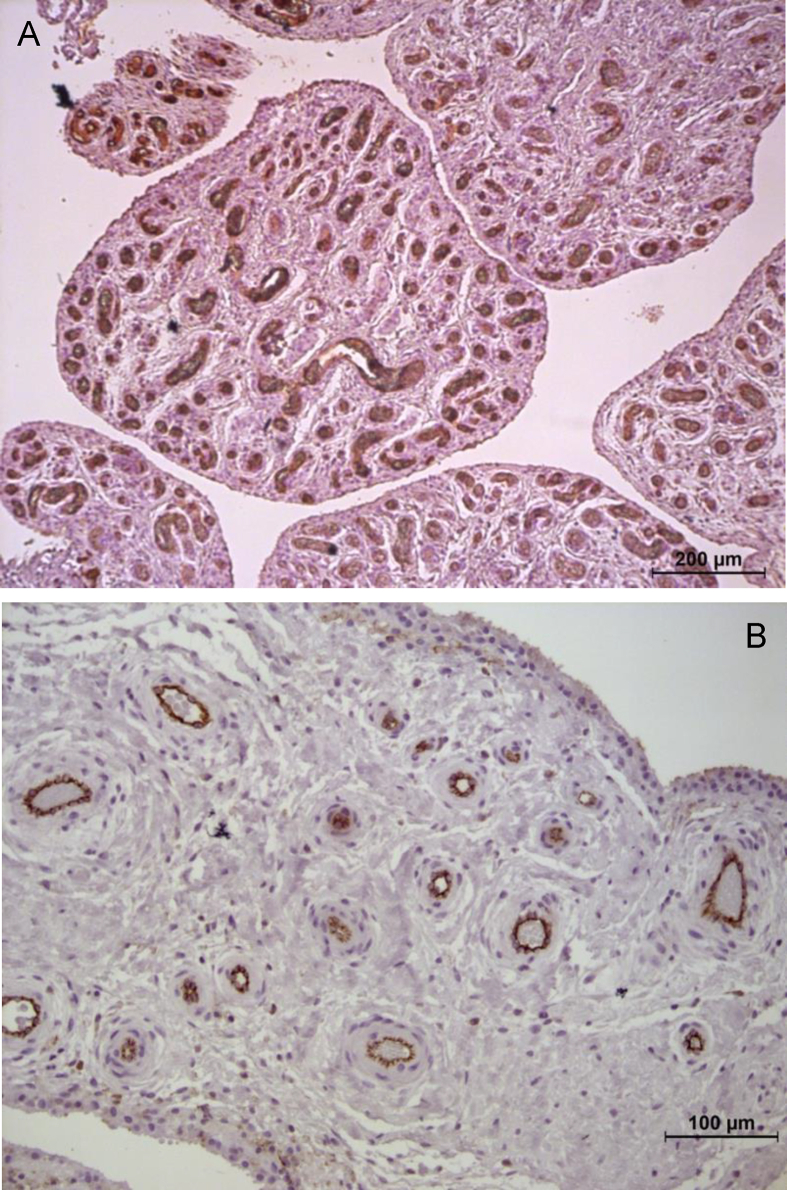
Difference of synovial hypervascularity, paraffine sections stained for CD31. A) hypervascularity from RCA group (magnitude 10×); B) Rotator Cuff Tear (RCT) group (magnitude 20×), vessels are identified by brown reaction product.

Several studies have suggested that epitope formation by bone or cartilage breakdown products might induce MCs activation via TLRs on MC membrane [22]. Following activation, MCs immediately degranulate their granule-stored mediators and start to synthesize new granules and mediators as a late response. These mediators include histamine, proteases, heparin, IL-6, TNFα, IL-1 and VEGF [16]. In addition, FcγRI is responsible for producing abundant TNFα from synovial MCs in response to aggregated Igg [15]. The release of these mediators could, at least in part, explain why patients affected by shoulder OA displayed marked synovial hypervascularity and higher levels of synovitis compared to controls (Fig. 4).

The present study has several limitations that warrant disclosures. Firstly, the design of the study does not permit to conclude on cause – effect relation, these findings suggest that MCs might play a role in the pathogenesis of clinical and histopathological expressions of OA in non-weightbearing large joints. The limited sample size made the present study underpowered. However, the strength of the study was that all patients in OA group were characterized by an eccentric OA due to RCA, therefore our results demonstrated that with the worsening of arthropathy the MCs infiltrate increases suggesting a potential role of MCs in RCA pathogenesis [23].

## 5 Conclusions

The present study demonstrated that patients affected by RCA were characterized by a higher number of MCs infiltrating the synovium. In addition, a positive correlation was found between MC number and radiological OA grade, synovitis grade and pain levels, while a negative correlation was found with functional scores even though the study was underpowered. Further studies are needed to clarify biochemical pathways beyond MCs activity in course of OA and possibly to better define the cause – effect relationship between MCs infiltration in synovium and expression of OA. Our findings open the way for the investigation of the role of MCs in OA, and the needed for new anti-neuroinflammatory and viscosupplementation therapy with addiction of drugs (i.e. Adelmidrol) acting by reducing mast cell activation.

## Author contributions

Conceptualisation, LF, AG; methodology, FA and CC; data curation and synthesis, FA and CC; writing—original draft preparation: LF and SM; writing—review and editing, LF and AG; supervision, AG; all authors interpreted the data, critically reviewed the work, made important contributions to the manuscript with their suggestions for improvement, approved the published version and agreed to be responsible for all aspects of the work. All authors have read and agreed to the published version of the manuscript.

## Role of the funding source

None.

## Declaration of competing interest

Nothing to declare.
